# Bioinspired radiative cooling coating with high emittance and robust self‐cleaning for sustainably efficient heat dissipation

**DOI:** 10.1002/EXP.20230085

**Published:** 2023-12-29

**Authors:** Yong Li, Yingnan Song, Hongye Zu, Feilong Zhang, Hui Yang, Wei Dai, Jingxin Meng, Lei Jiang

**Affiliations:** ^1^ Technical Institute of Physics and Chemistry Chinese Academy of Sciences Beijing People's Republic of China; ^2^ School of Future Technology University of Chinese Academy of Sciences Beijing People's Republic of China; ^3^ School of Materials Science and Engineering Nanyang Technological University Singapore Singapore; ^4^ Binzhou Institute of Technology, Weiqiao‐UCAS Science and Technology park Binzhou People's Republic of China

**Keywords:** Al alloy heat sink, cooling efficiency, heat dissipation, self‐cleaning, superhydrophobic

## Abstract

To overcome the overheating phenomena of electronic devices and energy components, developing advanced energy‐free cooling coatings with promising radiative property seem an effective and energy‐saving way. However, the further application of these coatings is greatly limited by their sustainability because of their fragile and easy contamination. Herein, it is reported that a bioinspired radiative cooling coating (BRCC) displayed sustainably efficient heat dissipation by the combination of high emittance and robust self‐cleaning property. With the hierarchical porous structure constructed by multiwalled carbon nanotubes (MWCNTs), modified SiO_2_ and fluorosilicone (FSi) resin, the involvement of the BRCC improves the cooling performance by increasing ≈25% total heat transfer coefficient. During the abrasion and soiling tests, the BRCC‐coated Al alloy heat sink always displays stable radiative cooling performance. Moreover, the simulation and experimental results both revealed that reducing surface coverage of BRCC (≈80.9%) can still keep highly cooling efficiency, leading to a cost‐effective avenue. Therefore, this study may guide the design and fabrication of advanced radiative cooling coating.

## Graphical abstract

Inspired by the lotus leaves, we present a bio‐inspired radiative cooling coating (BRCC) with hierarchical porous structures, consisting of multiwalled carbon nanotubes, modified SiO_2_ and fluorosilicone resin. The BRCC displayed sustainably efficient heat dissipation by the combination of high emittance and robust self‐cleaning property. It is expected to be a promising candidate for thermal management applications for power‐intensive energy components.

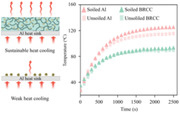

## 1 INTRODUCTION

With the rapid development of electronic devices and energy components toward for miniaturization, high levels of integration, and high power, removing excessive heat with high efficiency and stability is urgently required to upgrade the performance and lifetime of these devices.^[^ ^1^, ^2^ ^]^ Due to the constraints of compact space, size and environment, it is impossible to remove heat by accelerated convection. The heat sink without fan is preferred for cooling mini‐sized devices. Emitting heat via electromagnetic wave, radiative cooling has attracted great attention for the outstanding advantages such as less noise, less space and energy saving.^[^ ^3^, ^4^ ^]^ Aluminium (Al) alloy has been widely used as heat sinks because of its high thermal conductivity (≈270 W m^‐1^ K^‐1^), low thermal expansion and low density.^[^ ^5^ ^]^ However, the intrinsic low emittance of Al alloy (≤0.2) leads to the low radiative heat flux and may limit further applications. Thus, enhanced heat cooling by radiation may provide a promising avenue to improve heat cooling performance of heat sink.

In recent years, various advanced coatings have been developed to improve the emittance (*ε*) of Al alloy including anodic aluminium oxide (AAO) film,^[^ ^6^, ^7^, ^8^ ^]^ ceramic coating,^[^ ^9^, ^10^, ^11^, ^12^ ^]^ polymer‐based coating,^[^ ^13^, ^14^ ^]^ and organic–inorganic composite coating.^[^ ^15^, ^16^, ^17^, ^18^, ^19^ ^]^ For instance, Lee's group found that nano‐porous AAO with black sealing exhibited a high *ε* of 0.906.^[^ ^8^ ^]^ Mahadik et al. produced the Al_2_O_3_/SiO_2_ double‐layer coating with a high *ε* of ≈0.92.^[^ ^11^ ^]^ Interestingly, Yu and coworkers developed a planar PDMS cooling structure on the Al substrate with a high *ε* of 0.94, which can be utilized for all‐day radiative cooling.^[^ ^13^ ^]^ Despite extensive efforts, the radiative cooling materials are limited by complex fabrication processes or poor durability in real‐life applications. Most of these coatings inevitably suffer from the deposition of pollutants, leading to a dramatic decrease in the performance of radiative cooling.^[^ ^20^, ^21^ ^]^ Thus, it is highly desired to integrate self‐cleaning property with a radiative cooling coating.

In nature, the presence of a water film and pollutants on leaves can reduce gas exchange and sunlight transmittance. The self‐cleaning property of lotus leaves can enhance its efficiency of photosynthesis.^[^ ^22^ ^]^ When a water droplet falls on the lotus leaf, it showed the spherical shape and easily rolled off the surface taking away the adherent dirt particles, which called “lotus effect.”^[^ ^23^ ^]^ The self‐cleaning property of lotus leaves originates from the cooperative effect of micro/nano hierarchical structures and a wax layer. Inspired by lotus leaf, many different approaches have been developed to prepare superhydrophobic self‐cleaning surfaces.^[^ ^24^, ^25^, ^26^ ^]^ For instance, Jiang et al. prepared a stable superhydrophobic coating on copper surfaces.^[^ ^24^ ^]^ In addition, the robustness of these surfaces can be improved by integrating suitable nanoparticles such as carbon nanotubes (CNTs) owing to their high mechanical strength.^[^ ^27^, ^28^ ^]^ CNTs have played an indispensable role in thermal management,^[^ ^29^, ^30^, ^31^ ^]^ because of their high thermal conductivity and high emittance (≈0.99).^[^ ^32^, ^33^, ^34^, ^35^ ^]^ However, the dispersion of CNTs in solution is difficult due to its high surface energy, which is attributed to π–π interactions and van der Waals forces.^[^ ^36^ ^]^ The agglomeration of CNTs will increase the difficulty to obtain an interconnecting network and hinder phonon transport, adversely influencing the heat transfer performance of polymer nanocomposites.^[^ ^37^, ^38^ ^]^ The surface modification of filler particles is an efficient way to improve their dispersion to lower the thermal interface resistance. Hubmann et al. modified multiwalled carbon nanotubes (MWCNTs) via oxidative functionalization to improve the heat transfer performance of the polymer nanocomposites.^[^ ^39^ ^]^ Unique membrane structures endow coatings with controlled properties in both biological and artificial systems.^[^ ^40^ ^]^ It is easier to construct a heat transfer network in a polymer matrix by synergistic effect of multiple structures with different morphology and size.^[^ ^41^ ^]^ Thus, we wonder if the combination of robustness and self‐cleaning via the synergistic construction of MWCNTs and SiO_2_ can endow the radiative cooling coating with sustainably efficient heat dissipation.

**FIGURE 1:**
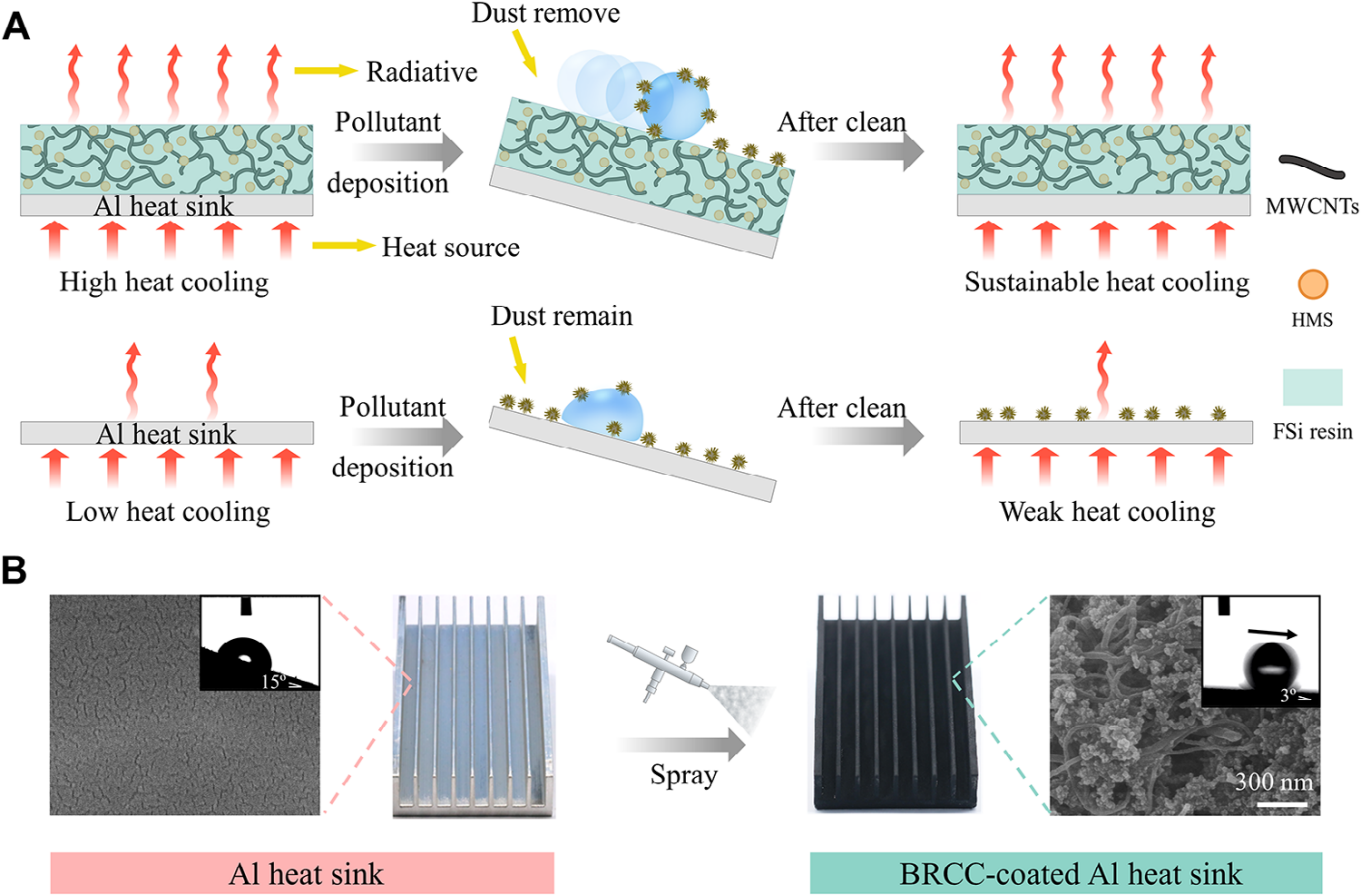
Design and fabrication of bio‐inspired radical cooling coating (BRCC). (A) Inspired by lotus leaf, the BRCC‐coated Al alloy heat sink was designed for effective and sustainable heat cooling by the combination of radiative heat dissipation and self‐cleaning property. In contrast, the surface temperature of the bare Al alloy heat sink increased due to pollutants still adhered even at a highly inclined plane (>10°). (B) The fabrication of the BRCC‐coated Al alloy heat sink by a simple and facile spraying approach. Insets show that water droplet can easily roll off the BRCC surface at a low sliding angle (≈3°), while adhere on the bare Al alloy even at a highly inclined plane (>10°).

Herein, we fabricate a bioinspired radiative cooling coating (BRCC) with high emittance and robust self‐cleaning, displaying sustainably efficient cooling performance. Hierarchical porous structure was constructed by good dispersion composites of MWCNTs, modified SiO_2_ and fluorosilicone (FSi) resin. Compared with the bare Al alloy heat sink, the BRCC‐coated one always increases ≈25% total heat transfer coefficient, resulting from the high heat dissipation of mixture of MWCNTs and the easy removal of deposited pollutant (Figure 1A). On one hand, the introduction of MWCNTs and modified SiO_2_ to the BRCC, the heat dissipation performance can be significantly increased due to high emittance. On the other hand, the BRCC‐coated Al alloy heat sink can keep stable cooling performance. During the soiling test, the pollutants can be easily taken away from the BRCC surface due to the low sliding angle of water droplet (≈3°) from the lotus effect (Figure 1A, top). In contrast, the pollutants still adhered on the bare one even at a highly inclined plane (>10°), leading to the increase of surface temperature (Figure 1A, bottom). As shown in the right part of Figure 1B, the BRCC is fabricated by spraying the mixture of the fibre‐like MWCNTs (Figure S1A) and spherical modified SiO_2_ (Figure S1B) on the Al surface. As shown in Figure S1C,D, the hierarchical porous structure of the BRCC are formed by mixing the fibre‐like MWCNTs and spherical modified SiO_2_, which were critical for trapping air to minimize the contact area between water and underlying coating. The experiment results show that the reducing surface coverage of the BRCC (≈80.9%) can still keep highly cooling efficiency, thereby leading to a cost‐effective avenue. Therefore, this study provides a promising avenue for effectively and sustainably thermal management for high‐power devices and equipment.

## 2 RESULTS AND DISCUSSION

### 2.1 Design and fabrication of BRCC

**FIGURE 2:**
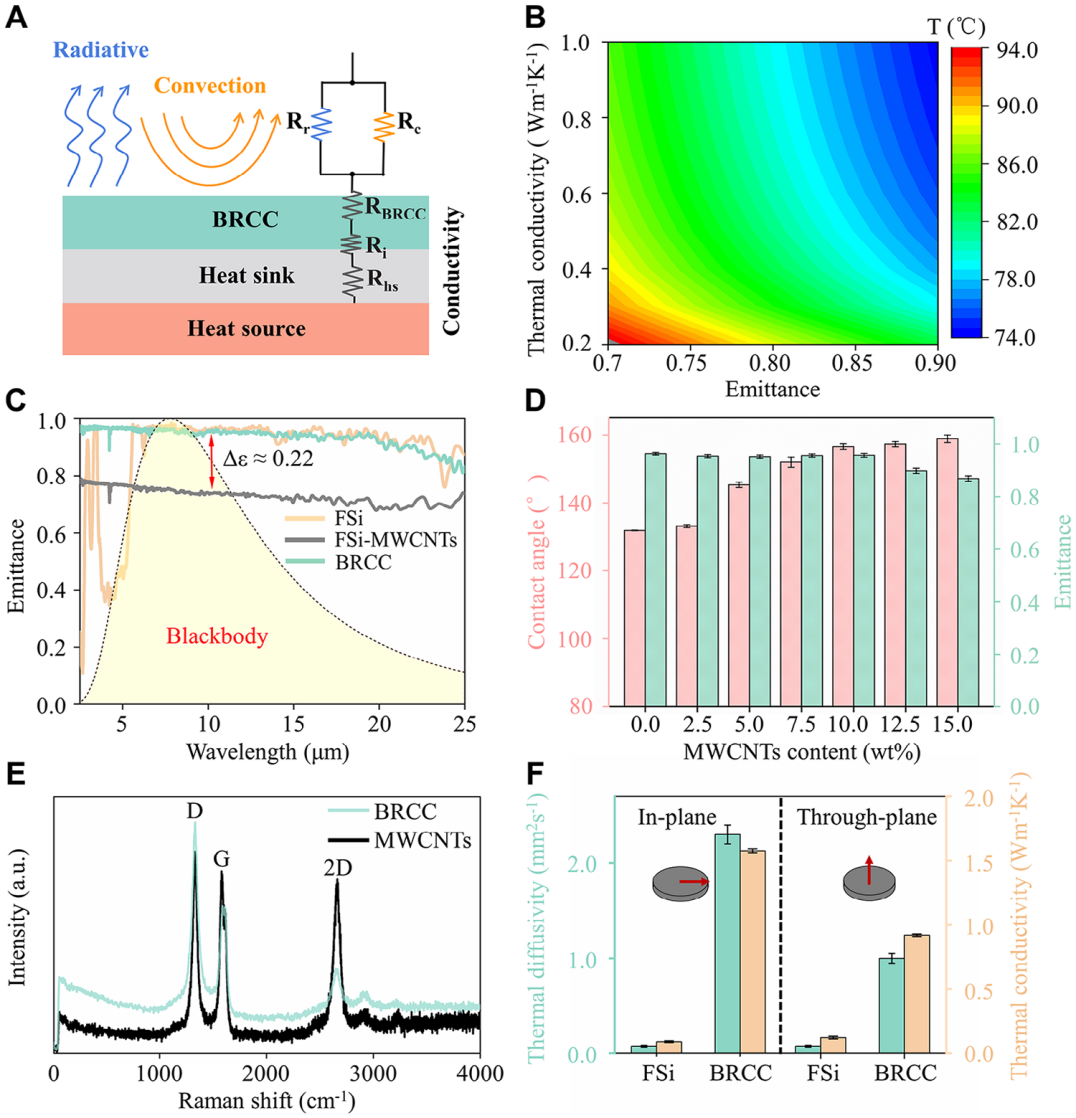
Thermal properties of the BRCC. (A) Schematic of thermal resistance of the BRCC‐coated Al alloy heat sink, showing the effect of factors (i.e. radiative, convection and conductivity) on heat cooling. (B) Surface temperature of the coating as a function of thermal conductivity and emittance. (C) The emittance of the coatings including the BRCC, FSi‐MWCNTs (the mixture of MWCNTs and FSi resin), and FSi resin. The blackbody radiation spectrum at 25°C. (D) Effect of the MWCNTs content on surface wettability and emittance. Three measurements were conducted, with the error bars representing the standard deviations. (E) Raman spectra of the BRCC and MWCNTs. (F) Thermal diffusivity and conductivity of the BRCC and FSi resin. Three measurements were conducted, with the error bars representing the standard deviations.

Generally, there are three ways for heat transfer, including heat conduction, heat convection, and heat radiation. For the heat sink without fan, heat convection is considered as a fixed value. To obtain effective and sustainable heat cooling performance in heat sink, it is necessary to appropriately combine high emittance with high thermal conductivity. As shown in Figure 2A, the influence of different factors on radiative cooling performance has been simplified in the form of thermal resistance. Firstly, the introduction of BRCC should improve surface emittance of Al alloy heat sink. Then, the BRCC should also have high thermal conductivity, which is benefit for the decrease of thermal resistance of the BRCC‐coated Al substrate. In contrast, low thermal conductivity of BRCC will suppress heat transfer and cause heat accumulation, revealed by the simulation result of thermal conductivity and emittance (Figure 2B).

The key of our bioinspired strategy lies in the introduction of hierarchical structures on the surface of heat sink. The BRCC was fabricated on Al alloy substrates by spraying the mixture of HMDS modified SiO_2_ nanoparticles (denoted as HMS), MWCNTs and FSi resin (details in Methods). There are hydrophobic interactions and hydrogen‐bonding interactions between MWCNTs, modified SiO_2_ and fluorosilicone (FSi) resin. On one hand, there are hydrophobic interactions between the methyl group on the surface of the modified SiO_2_ and the fluorine group of FSi resin. On the other hand, hydrogen bonds were formed between the residual hydroxyl group of the hexamethyldisilazane‐modified SiO_2_ nanoparticles and the hydroxyl group on the surface of the MWCNTs. In addition, the aminoester bonds were formed between the MWCNTs and FSi resin too, which can be ascribed to the crosslinking reactions between the hydroxyl groups on MWCNTs and the isocyanate groups of the curing agent. Figure 2C shows *ε* for different coatings and the blackbody radiation spectrum at 25°C. To achieve excellent emittance for heat dissipation, the 901 FSi resin was selected as the hydrophobic adhesive for fabricating the BRCC, because it exhibits a high emittance of ≈0.96 at 8–13 µm (Figure 2C, orange curve) and excellent resistance to aging.^[^ ^42^ ^]^ As the increment of MWCNTs contents to 15 wt%, the lower infrared emittance (≈0.84) and the higher WCA (152.5 ± 2.6°) can be observed due to apparent surface cracks from poor dispersion of MWCNTs in FSi matrix (Figure 2C, grey curve, Figures S2 and S3). To simultaneously meet our demand for high emittance and superhydrophobicity, 5 wt% HMS nanoparticles were employed for improving the MWCNTs dispersion and the BRCC hydrophobicity (Figure S4). FT‐IR spectra of HMS in Figure S5, shows that the new peaks at 2923 and 2854 cm^−1^ ascribed to the stretching and bending vibrations of Si‐(CH_3_)_3_ groups.^[^ ^43^ ^]^ The continuous BRCC from HMS introduction exhibited a high *ε* value of ≈0.96 over thermal infrared wavelengths (2.5–25 µm) (Figures S3 and 2C, green curve), which is more beneficial for heat cooling. As shown in Figure 2D, the WCAs and emittance of the FSi resin is 103.7 ± 0.2° and 0.96, respectively. With the MWCNTs contents varying from 2.5% to 10%, the WCAs of the BRCC increase from 106.3 ± 0.9° to 153.1 ± 1.6° (Figure 2D), while the corresponding emittance is always higher than 0.95 from hierarchical porous structure of the BRCC. Even in a wide range of the incident angles from 60° to 60° (Figure S6), surface emittance of the BRCC is always higher than 0.91. Raman spectra (Figure 2E) show intense peaks of the lattice quantization of MWCNTs (D‐band at ≈1332 cm^−1^ and G‐band at 1577 cm^−1^) and retained in the BRCC. Further increasing the MWCNTs content to 12.5%, the BRCC emittance began to decrease (≈0.90) due to the appearance of fine cracks (Figure 2D). Thus, HMS/MWCNTs‐10 was selected for the following fabrication of the BRCC, which displays high emittance and superhydrophobic self‐cleaning property.

In addition, thermal conductivity is also important factor for heat dissipation of Al substrates. Compared with FSi resin (0.12 W m^‐1^ K^‐1^), the BRCC shows enhanced thermal conductivity from both through‐plane (0.90 W m^‐1^ K^‐1^) and in‐plane (2.10 W m^‐1^ K^‐1^), displaying ≈500% and 1500% enhancement in Figure 2F. The apparent thermal anisotropy of the BRCC may come from aligned parallel of the MWCNTs in‐plane direction of FSi resin.^[^ ^44^, ^45^, ^46^ ^]^ In addition, the inclusion of HMS nanoparticles is benefit for the high‐quality dispersion of the MWCNTs into FSi matrix, thereby facilitating the phonon transfer and minimizing interfacial thermal resistance. Thus, the BRCC not only has low thermal resistance in the through‐plane direction but also spreads the hot‐spot heat rapidly.

### 2.2 Anti‐soiling property

**FIGURE 3:**
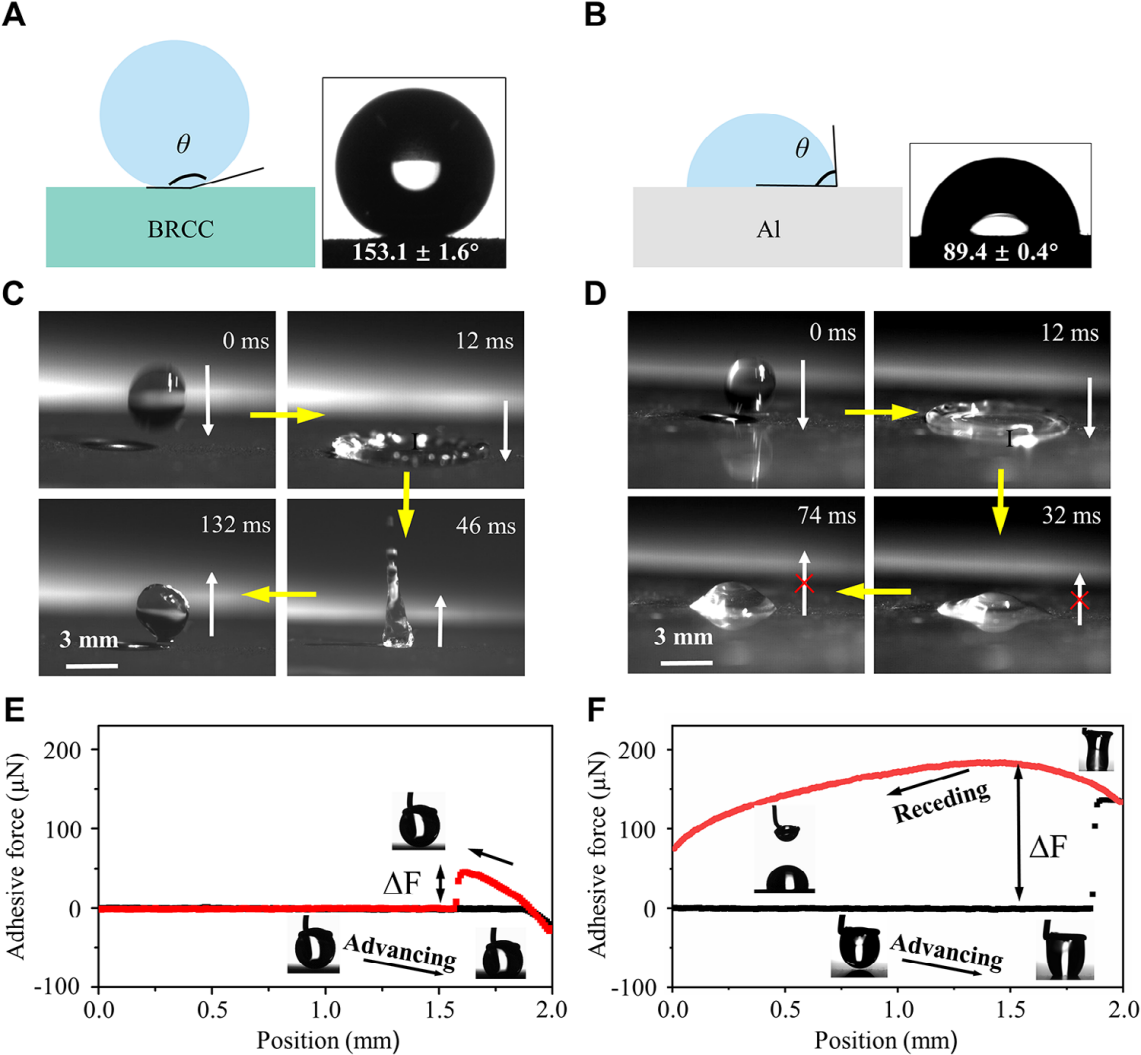
Surface wettability of the BRCC‐coated and bare Al alloy plate. The WCAs of (A) the BRCC‐coated and (B) the bare Al alloy plate. The bounce behaviour of water droplet on (C) the BRCC‐coated and (D) the bare Al alloy plate. Force‐distance curves and corresponding optical images of (E) the BRCC‐coated and (F) bare Al alloy plate.

Self‐cleaning property (e.g. anti‐soiling) is an important factor of radiative coolers for practical applications because it may relieve the degradation issue of heat transfer from surface contamination.^[^ ^47^, ^48^ ^]^ The as‐fabricated BRCC with hierarchical structures, exhibits robust, lotus leaf‐like superhydrophobic properties. After endowing the BRCC (Figure 3A), the WCA and the roughness (*Ra*) of the BRCC‐coated Al alloy plate was 153.1 ± 1.6° and ≈40 nm (Figure S7). Moreover, the anti‐soiling property of the BRCC was evaluated as shown in Movie S1 and Figure S8 When dropping a water droplet (≈10 µL) on the BRCC, it rapidly rolled off with a low sliding angle (SA) of ≈3° (≈244 ms). In contrast, the WCA of the bare Al alloy plate was 89.4 ± 0.4° (Figure 3B). During the water impacting test, the water droplet underwent a “spreading‐reaction” stage and bounced up several times before residing on the BRCC (Figure 3C and Movie S2). In contrast, the water droplet impacted and anchored on the Al alloy surface (Figure 3D and Movie S3). Moreover, there was nearly no deformation of water droplet on the BRCC‐coated Al alloy plate, revealed by a low adhesive force of 40.6 ± 2.7 µN (Figure 3E). When a water droplet left the bare Al alloy plate, it displays a large extent of shape distortion with a high adhesive force of larger than 191.7 ± 7.2 µN (Figure 3F). Similarly, as shown in Figure S9, the smooth FSi resin was highly adhesive to water (larger than 156.3 ± 0.7 µN). When a water droplet left the FSi coating, it underwent a large extent of shape distortion. The stretch force increased gradually until the water droplet broke. Thus, the BRCC may endow the substrates with excellent superhydrophobicity and low water adhesion.

**FIGURE 4:**
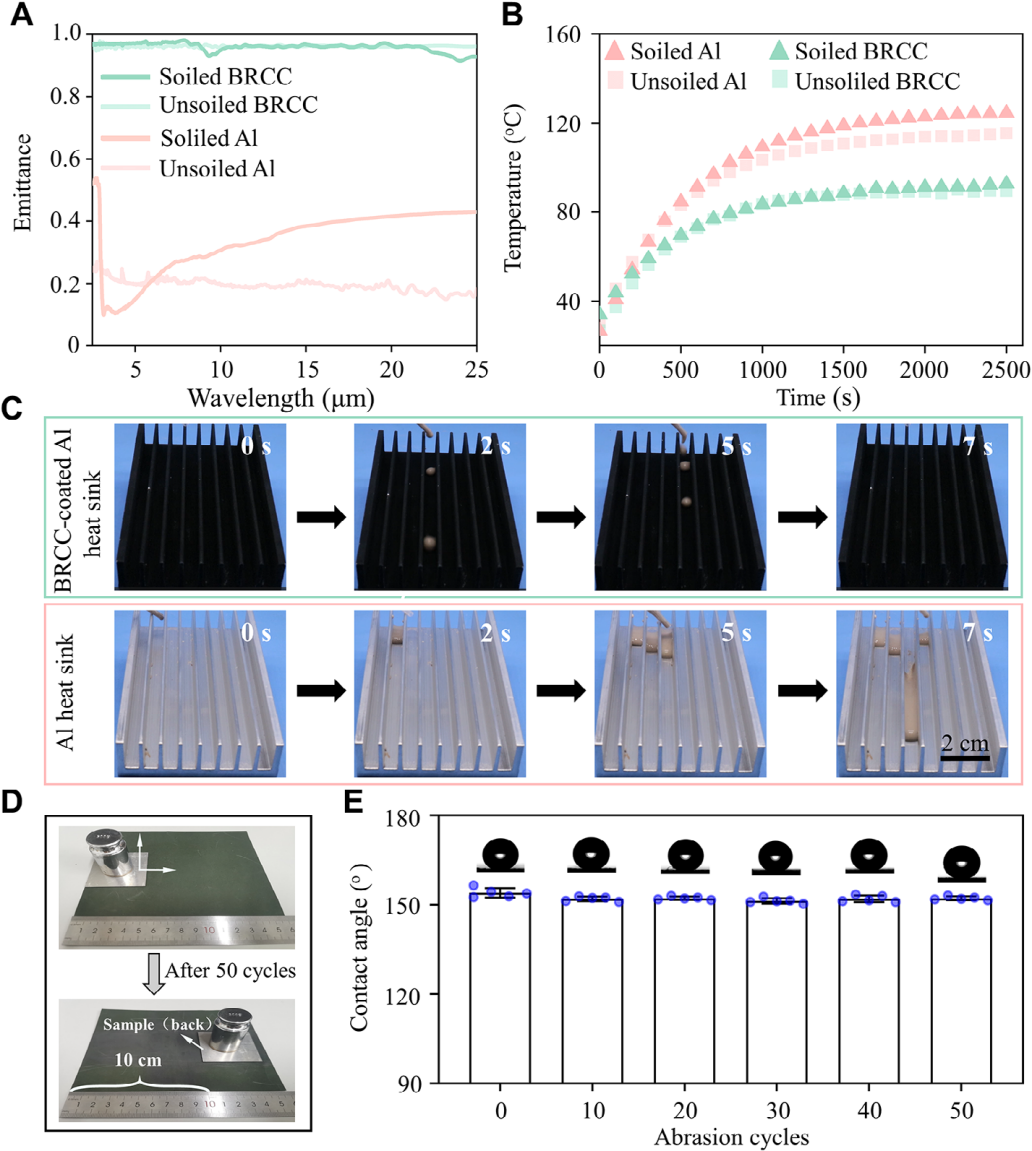
Robust self‐cleaning performance of the BRCC‐coated Al alloy surface. (A) Comparison of *ε* value of BRCC and bare Al alloy surface before and after soiling test. (B) The transient temperature curve before and after soling, indicating that the BRCC shows more stable heat cooling than that of bare Al alloy. (C) The viscous mud rapidly rolls off from the BRCC‐coated Al alloy heat sink, showing its excellent self‐cleaning performance. In contrast, the mud adhered on the bare one. The tilted angle was ≈10°. (D) The abrasion test for the BRCC. (E) Effect of abrasion cycles on the wettability of the BRCC.

As a proof of concept, the anti‐soiling performance of the BRCC‐coated and bare Al alloy plate was compared. Taking the dust powder of coal ash and TiO_2_ (w/w, 1/2) as a model contaminant, the dust on the BRCC‐coated Al alloy plate could easily be removed by water droplets (Figure S10). In contrast, water droplet was pinned on the bare one, and most of the contaminant remained. To explore the anti‐soiling effect, we soiled the samples by dripping the low viscous mud (coal ash 50%, the white pigment of TiO_2_ 10%, water 30%) and then measured the ε of the BRCC and Al alloy plate. Compared with the unsoiled BRCC, the soiled one shows only ≈0.2% decrease of emittance. By contrast, the significant change of emittance occurred for bare Al alloy (from 0.194 to 0.265) from the mud adhesion (Figure 4A). To further show excellent ability to reduce the accretion of pollutant, we dripped low viscous mud onto the BRCC‐coated Al alloy heat sink and recorded the temperature difference (Δ*T*) between the unsoiled samples and soiled ones (Figure 4B,C and Movies S4 and S5). The soiled process has slight effect on the cooling performance of the BRCC‐coated Al alloy heat sink, revealed by a small increase of Δ*T* from ≈88.7°C to 91.2°C. In contrast, a relatively large effect on the bare one, indicated by a large increase of ≈10.0°C. Even in high humidity or high temperature, the Al alloy heat sink with BRCC still kept self‐cleaning performance (Movie S6) and exhibited stable cooling performance (Figure S11 and Movie S7). Therefore, these results show that the as‐prepared BRCC endows the Al alloy substrate with efficient and stable heat cooling performance.

### 2.3 Durability of the BRCC

To apply in a practical and harsh scene, the influence of harsh environments (wide pH range, liquid impact and abrasion) on self‐cleaning property was explored and durability of the BRCC was estimated. After dropping liquid droplets (pH value from 1 to 13), they always displayed the stable WCAs of over 150° on the BRCC (Figure S12), suggesting the tolerance of a wide pH range. Moreover, the durability of the BRCC was evaluated by employing cross‐cut test and sandpaper abrasion test.^[^ ^49^ ^]^ Figure S13 shows the schematic illustration of the cross‐cut test based on standard ISO 2409. After scratching, deeply vertical cross‐scratches can be observed on the BRCC, exposing the underlying Al substrate. Only a few squares of the lattice (<3%) were detached from the Al substrate, indicating the firm attachment of the BRCC (classification 1).^[^ ^49^ ^]^ After cross‐cut test, water droplet remained spherical on the surface of the BRCC. During the sandpaper abrasion test, one‐cycle abrasion was regarded by vertically and horizontally moving the BRCC for 10 cm each on the sandpaper (1000 grit) under a weight of 200 g. Even after 50‐cycle abrasion, it still maintained a high WCA of ≈152° and a low SA of ≈6° (Figure 4D,E). Moreover, water flush could easily bounce off the BRCC without any damages (Movie S8). Therefore, the BRCC displayed the excellent durability under harsh conditions.

### 2.4 Heat dissipation performance

To evaluate radiative heat transfer, the surface temperature of the BRCC‐coated Al alloy plate and bare Al alloy plate were tested on the same hot stage and recorded by infrared imaging instrument. It should be noted that the temperature distribution by the infrared camera is not the real temperature but reflects on the degree of radiation heat transfer. The higher emittance of the object, the more radiative heat, and the higher temperature measured. After heating for different times (0, 20, 40, and 60 s), the infrared images of the samples were presented in Figure S14. Compared with the bare Al alloy plate in blue colour, the BRCC‐coated one changed quickly from blue to red colour in a short time. The BRCC displayed a much increment of apparent surface temperature with 55°C after heating 60 s. Thus, the radiative heat transfer performance of Al alloy plate is significantly strengthened by introducing the BRCC with high thermal conductivity and high emittance.

**FIGURE 5:**
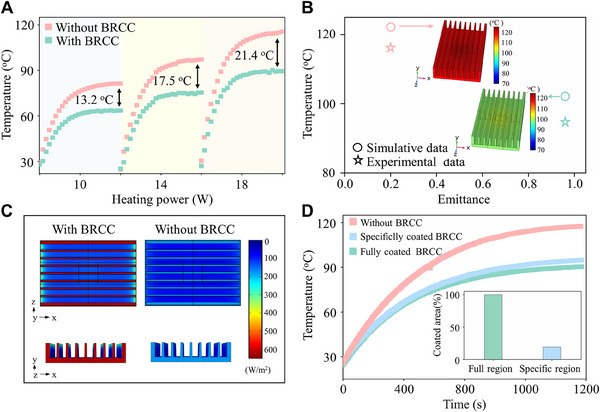
Comparison of heat dissipation performance of Al alloy heat sinks with BRCC and without BRCC. (A) Transient temperature curves of Al alloy heat sinks with BRCC and without BRCC under different heating powers (i.e. 10, 14, and 18 W). (B) Comparison of simulation results and experimental data at 18 W. Insets show the corresponding temperature fields. (C) Simulation of radiative heat flux of distribution from subduction and side view. (D) Transient temperature curves of Al alloy heat sinks with different modified styles of the BRCC including bare, specific BRCC and full BRCC. Inset shows that there is ≈80.9% reduction in the coating area for the specific BRCC compared with the full BRCC.

To further evaluate heat dissipation performance, the temperature distribution and heat transfer performance of the BRCC‐coated and bare Al alloy heat sink were explored by experimental verification and numerical simulation (Figure 5). Figure 5A shows that temperature gradients are formed on the Al alloy heat sink (with and without BRCC). Significantly, the equilibrium temperature of the BRCC‐coated Al alloy heat sink was decreased by 13.2°C, 17.5°C, and 21.4°C at heating powers of 10, 14, and 18 W. Correspondingly, the simulation shows that the BRCC can reduce the maximum temperature of the heat sink by 12.9°C, 16.6°C, and 20.0°C when the heating power is 10, 14, and 18 W. Taking the heating power of 18 W as an example, the total heat transfer coefficient (*U*) and thermal resistance (*R_T_*) of the BRCC‐coated Al alloy heat sink were increased by ≈25% and decreased by ≈19%, indicating that the introduction of BRCC greatly improved heat transfer performance of Al alloy heat sink (More details in Table S1). The equilibrium temperatures at 18 W from simulation are slightly higher than experimental results (Figure 5B), probably ascribing to the errors from numerical simulation, temperature measurement and environmental disturbance.^[^ ^34^ ^]^ The temperature of the BRCC‐coated Al alloy heat sink was much lower than that of bare one, confirming the excellent heat dissipation of the BRCC. To investigate thermal properties of the BRCC‐coated Al alloy heat sink, the simulations of the radiative heat flux distribution from different viewing angles were made at a heating power of 18 W (Figure 5C and Figure S15). Compared with the bare Al alloy fin, radiative heat flux of the BRCC‐coated Al alloy fin was greatly increased. Moreover, the peripheral surface of the BRCC‐coated Al alloy fins exhibited the highest radiative heat flux due to the largest ambient view factor,^[^ ^34^ ^]^ indicating that the BRCC‐coated outer surface of fins (red part) mainly play a great role in the heat flux. To verify simulation results, the Al alloy heat sink fin was selectively coated by BRCC (red part) and utilized for performing the heat dissipation test (Figure S16). Compare with the BRCC‐coated Al alloy heat sink with full region, the temperature increment of that with specific region only 4.1°C (Figure 5D). Even if only the outer surface of Al alloy fins was coated with the BRCC, it still keeps excellent radiative cooling performance. More importantly, the area of coated BRCC Al alloy heat sink reduced 80.9%. Thus, this strategy is benefit for the cost reduction and efficiency improvement in practical application.

## 3 CONCLUSIONS

In summary, the BRCC‐coated Al alloy heat sink was fabricated by a facile spraying approach, displaying excellent passive heat dissipation and robust self‐cleaning property. The efficient radiative cooling performance of as‐prepared heat sink originates from the synergistic effect of high emittance and high thermal conductivity. Significantly, the BRCC is beneficial for alleviating the heat dissipation efficiency degradation caused by surface contamination. In addition, the cross‐cut and sand‐abrasion tests reveal the robustness of the BRCC coating, greatly improving the stability of heat dissipation. Simulation results guide us to selectively coat BRCC on Al alloy heat sink, which is benefit for reducing the production cost and improving procedure efficiency. Therefore, this study holds huge potentials for thermal management applications in high‐power energy components.

## 4 EXPERIMENTAL SECTION

### 4.1 Preparation of HMS nanoparticle

We prepared HMS nanoparticles according to previous reports.^[^ ^43^ ^]^ In brief, SiO_2_ nanoparticles (500 mg) were first dispersed into absolute ethyl alcohol (100 mL) and sonicated for 15 min. Then, HMDS (20 mL) was dropwise added into the above solution. Later, the mixture was stirred at 60°C for 8 h and placed into sealed beakers for 24 h. Further, the solution was centrifuged at 9000 rpm for 10 min and washed with ethanol for three times. Finally, HMS were successfully obtained after vacuum drying at 80°C for 24 h.

### 4.2 Fabrication of the BRCC

The BRCC was deposited on the substrates (Al alloy plate or Al alloy heat sink) by a facile spraying method. Taking the BRCC with 10 wt% MWCNTs as an example, 0.33 g HMS and 0.66 g MWCNTs was firstly dispersed in a mixture of ethyl acetate and butyl acetate (1/1, v/v), followed by ultrasonic sonication for 15 min. 6.6 g FSi resin containing 901 FSi resin and curing agent N3390 (10/1, w/w) was gradually added to the above mixture solution and vigorously stirred for 10 min. Finally, the BRCC with a thickness of ≈100 µm was prepared by employing the spraying method (an air pressure of 0.2 MPa, Figure S17 and Movie S9).

### 4.3 Measurement of the in‐plane and through‐plane thermal diffusivity and conductivity

To evaluate the thermal conductivity, the samples were measured by thermal constants analyzer (Hot Disk) with reference resistance at 6.922 Ω, heating power at 30 mW and measurement time at 20 s. Thermal conductivity can be acquired from the equation *λ* = *α ×C_p_ × ρ*, where *α* is the thermal diffusivity, *C_p_* is the heat capacity and *ρ* is the density. The in‐plane and through‐plane thermal diffusivities of the BRCC and FSi resin were obtained at a constant pressure using a laser flash method (LFA 467, Netzsch, Germany), as shown in Figure S18. The exposed sample surface is irradiated with a very short laser pulse and the temperature rise is measured on the opposite side of the sample by an IR detector. The upper surface of through‐plane measured sample was completely exposed to the IR detector, but the in‐plane measured sample was sheltered by a metal cover with only four windows to be detected. Therefore, the thermal diffusivity in the in‐plane direction can be gained. The specific heat of the BRCC and FSi resin were measured using differential scanning calorimetry (DSC, Q2000, TA Instruments, USA) according to the ISO 1135‐4: 2005.

### 4.4 Measurement of adhesive force

To evaluate the adhesion forces of water, a high‐sensitivity microelectromechanical balance system (DCAT 11, Data Physics, Germany) was employed. For each value, three measurements of per sample were performed and average value was obtained.

### 4.5 Experimental condition for evaluating heat transfer performance

To evaluate heat transfer performance, the Al alloy heat sink with nine fins was employed (a length of 100 mm, a spacing of 5 mm, and a height of 13.5 mm). The self‐made thermal dissipation measurement system is shown in a previous report.^[^ ^50^ ^]^ In brief, a cartridge heater (10 mm × 10 mm) was used as the heat source, providing the heating power (i.e. 10, 14, and 18 W). To accurately measure the temperature, a K‐type thermocouple was attached directly to the middle of the Al alloy heat sink by heat conducting glue. A 4‐channel K thermometer SD logger (a resolution of ± 1°C, AZ instrument) was used to record the temperature of heat sink at an interval time of 2 s. If temperature change of the heat sink was lower than 0.2°C, we considered that it reached a steady state. All the experiments were performed under a stable and wind‐less environment.

### 4.6 Thermal simulation

To evaluate radiative heat dissipation of the Al alloy heat sink, COMSOL software was employed for simulating the distribution of temperature field and radiative heat flux. Three factors were considered including temperature, total heat transfer coefficient and thermal resistance. During the modelling process, thermal conduction and radiation are coupled in the model, and natural convection is considered as a boundary condition with a given heat transfer coefficient. To simplify the calculation, a half heat sink is taken as the computational domain. Hexahedral mesh with a number of 14,200 is selected. The detailed process of thermal simulation was mentioned in a previous report.^[^ ^34^ ^]^ The total thermal resistance and total heat transfer coefficient are displayed in the following equations.

(1) $$R_{T}=\left(T_{bmax}-T_{\mathrm{amb}}\right)/P$$

where *R_T_* is the total thermal resistance; *P* is the total dissipated power of a heat sink; *T_bmax_* is the maximum temperature of the heat sink; and *T_amb_* is the ambient temperature. At a certain dissipated power, if the thermal resistance is smaller, the temperature rise would be smaller, which means the heat sink displays better heat transfer performance.

(2) $$U=P/U\left(T_{h}-T_{amb}\right)$$

where *U* is the total heat transfer coefficient; *A* is the surface area for heat transfer; *T_h_* is the average temperature on the outer surface of heat sink.

## CONFLICT OF INTEREST STATEMENT

The authors declare no conflicts of interest. Jiang Lei is a member of the *Exploration* editorial board.

## Supporting information

Supporting Information

Supporting Information

Supporting Information

Supporting Information

Supporting Information

Supporting Information

Supporting Information

Supporting Information

Supporting Information

Supporting Information

## Data Availability

All data of this work are present in the article and the Supporting Information. The other data that support the findings of this work are available from the corresponding author upon reasonable request.
