# Supplementary material for: Bioinspired radiative cooling coating with high emittance and robust self‐cleaning for sustainably efficient heat dissipation
Source: Exploration (Beijing). 2023 Dec 29;4(3):20230085. doi: 10.1002/EXP.20230085 (PMC11189568; doi:10.1002/EXP.20230085)
Supplement: Supplementary file 10 — Supporting Information [file EXP2-4-20230085-s005.pdf]

Electronic Supplementary Information

Bioinspired radiative cooling coating with high emittance and robust self-cleaning for sustainably efficient heat dissipation

Yong Li‡[1]| Yingnan Song‡[1]| Hongye Zu[1]| Feilong Zhang[1,2,5]| Hui Yang[1]| Wei Dai[1]| Jingxin Meng[1,2,3]|Lei Jiang[1,2,4]

[1]Technical Institute of Physics and Chemistry, Chinese Academy of Sciences, Beijing 100190, P. R. China.

[2]University of Chinese Academy of Sciences, Beijing 100049, P. R. China.

[3]Binzhou Institute of Technology,Weiqiao-UCAS, Science and Technology park, Binzhou 256606, P. R. China.

[4]School of Future Technology, University of Chinese Academy of Sciences, Beijing, P. R. China.

[5]School of Materials Science and Engineering, Nanyang Technological University, Singapore 639798, Singapore.

***Corresponding authors.** E-mail: mengjx628@mail.ipc.ac.cn; huiyang@mail.ipc.ac.cn; cryodw@mail.ipc.ac.cn

† Y. L., Y. S. contributed equally to this work

Contents:

1. Supplementary Notes

1.1 Materials 4

1.2 Characterization 5

2. Supplementary Figures

Figure S1 Surface morphologies of MWCNTs, HMS and BRCC..........................6

Figure S2 Effect of MWCNTs content on surface wettability and emittance of FSi-MWCNTs...7

Figure S3 Optical images of Al alloy plates coated with FSi-MWCNTs and BRCC...8

Figure S4 Surface wettability of FSi, FSi-MWCNTs and BRCC...........................9

Figure S5 FT-IR spectra of HMS nanoparticles..10

Figure S6 Directional emittance of the BRCC at different angles.........................11

Figure S7 AFM images of the BRCC..12

Figure S8 Water droplet rapidly rolls off the BRCC with a low sliding angle of ca. 3°...13

Figure S9 Force-distance curves and corresponding optical images of the FSi-coated Al alloy plate..14

Figure S10 Comparison of surface dust removement *via* water flushing 15

Figure S11 The high temperature resistance of the Al alloy heat sink with BRCC...16

Figure S12 Chemical stability of the BRCC...17

Figure S13 Robust wettability of the BRCC...18

Figure S14 Comparison of thermal property between the BRCC-coated and bare Al alloy plates...19

Figure S15 Simulation of radiative heat flux of heat sinks...................................20

Figure S16 The experimental set-up for temperature measurement of Al alloy heat sink with full BRCC and specific BRCC..................................21

Figure S17 Spraying the BRCC on Al alloy heat sink...22

Figure S18 Schematic of the thermal diffusivity measurement process................23

3. Supplementary Table

Table S1 Simulation of heat transfer property of the heat sink.............................24

4. Supplementary Movies

Movie S1: Water droplet rolls off the BRCC-coated Al alloy plate.......................25

Movie S2: Dynamic impact process of water droplet on the BRCC.....................25

Movie S3: Dynamic impact process of water droplet on the bare Al alloy plate...25

Movie S4: Viscous mud rolls off the BRCC-coated Al alloy heat sink.................25

Movie S5: Viscous mud adheres on the bare Al alloy heat sink............................25

Movie S6: Self-cleaning performance in high humidity..25

Movie S7: Self-cleaning performance in high temperature...................................25

Movie S8: Flushing process on the BRCC after 50-cycle abrasion.......................25

Movie S9: Spraying the BRCC on Al alloy heat sink..25

1. Supplementary Notes

1.1. Materials

Hydroxylated multiwall carbon nanotubes (MWCNTs) (diameter: 10-20 nm, length: 10-30 μm) were purchased from Nanjing XFNANO Materials Tech Co., Ltd. Hexamethyldisilazane (HMDS) (AR, 98%) and ammonia solution (AR, 98%) were purchased from Shanghai Macklin Biochemical Co., Ltd. Ethyl acetate, butyl acetate and absolute ethyl alcohol was purchased from Guangzhou chemical reagent factory. Deionized water was prepared by Purification equipment of Millipak® Eppress 40. SiO_2 nanoparticles with a diameter of ca. 15 nm were purchased from Hongde Nanomaterials Co., Ltd. Fluorosilicone (901 FSi) resin was purchased from Shanghai Fukang New Material Technology Co., Ltd. China. N3390 curing agent was purchased from Bayer. Polished aluminum (Al) alloy plate and Al alloy heat sink was both purchased from Wanshun electronics Co., Ltd.

1.2. Characterization

Surface morphology of the BRCC was characterized by scanning electron microscope (SEM, JEOL, JSM-5900) and atomic force microscope (AFM, NT-MDT Prima, Bruker Dimension Edge, Germany). FT-IR spectra were measured using a Nicolet iS50 FT-IR spectrometer (Thermo Electron Madison, USA) and Raman spectra were collected using laser Raman spectrometer (Invia-ReFlex, UK). Surface wettability was characterized by static water contact angle (WCA) and sliding angle (SA) (Powereach JC2000D1). Adhesive force of water droplet on the coating was evaluated by fixing a water droplet (5 μL) on a copper spiral in a microelectronic balance system (DCAT11, Dataphysics, Germany). Dynamic impact of water droplet was recorded by a high-speed camera equipped with a Nikon camera lens. The microscope image of the coatings was observed by using an optical microscope (Ti-E, Nikon, Japan). Mud soiling test was explored by dripping low viscous mud on the samples, then dried under infrared heat lamp. Thermal diffusivity and specific heat of the BRCC and FSi resin were obtained at a constant pressure using a laser flash method (LFA 467, Netzsch, Germany). Thermal conductivity can be acquired from the equation $\lambda = \alpha \times Cp \times \rho$, where α is the thermal diffusivity, Cp is the heat capacity and ρ is the density. The emissivity (λ) was measured on a Bruker TENSOR 27 FTIR Spectrometer equipped with an integrating sphere (A562-G/Q).

2. Supplementary Figures

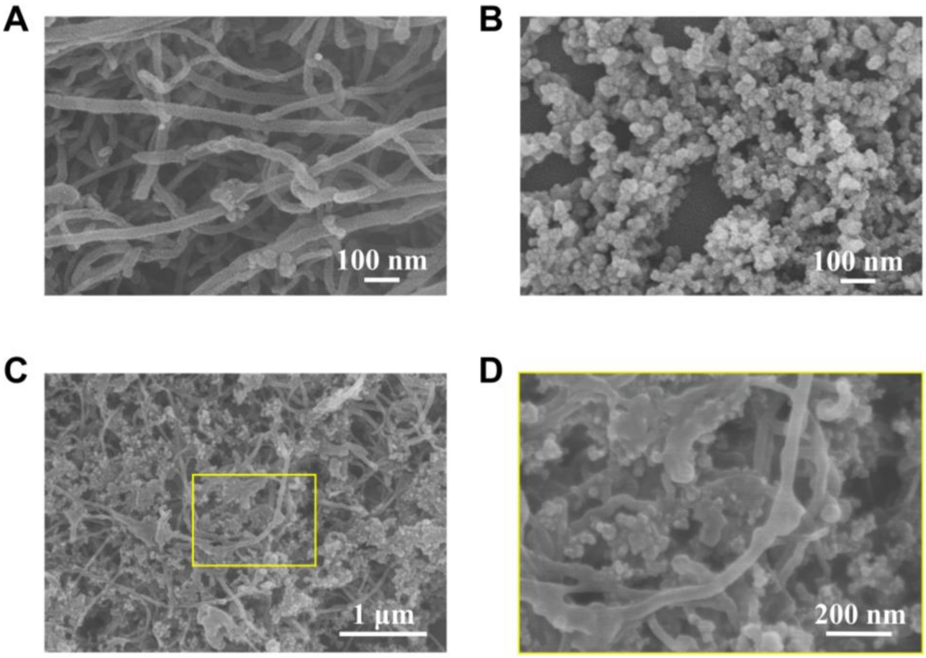

Figure S1 Surface morphologies of MWCNTs, HMS and BRCC. A) SEM image of the MWCNTs (the diameter of ca. 10-20 nm). B) SEM image of the HMS (the diameter of ca. 15 nm). C) SEM and D) enlarged SEM images of the BRCC with hierarchical porous structure.

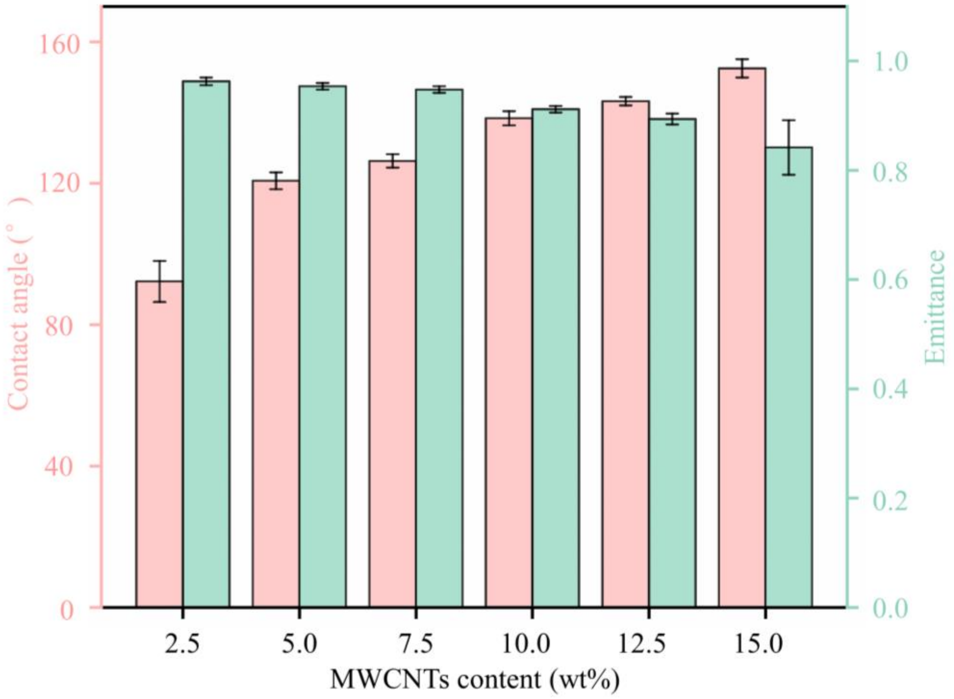

Figure S2 Effect of MWCNTs content on surface wettability and emittance of FSi-MWCNTs. The error bars represent the standard deviations.

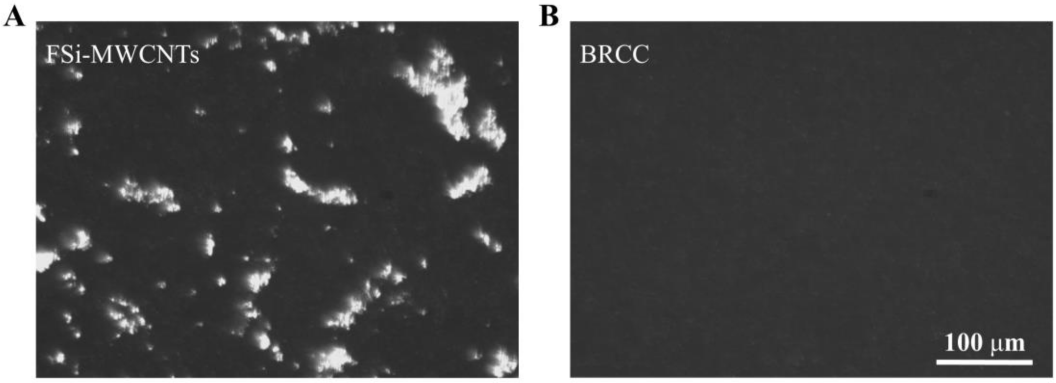

Figure S3 Optical images of Al alloy plates coated with A) FSi-MWCNTs and B) BRCC.

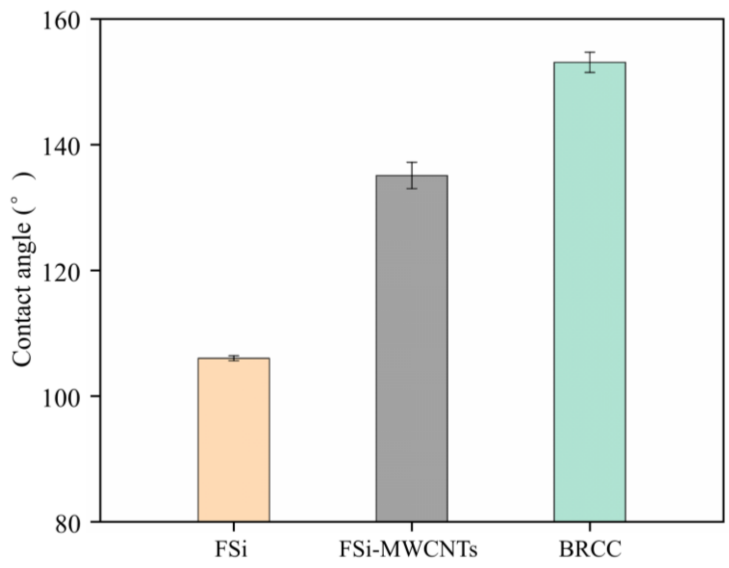

Figure S4 Surface wettability of FSi, FSi-MWCNTs and BRCC.

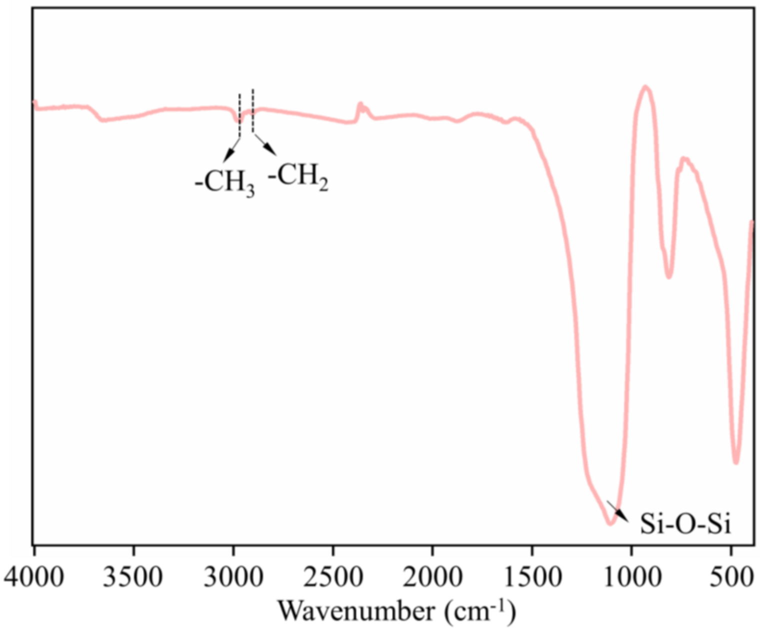

Figure S5 FT-IR spectra of HMS nanoparticles.

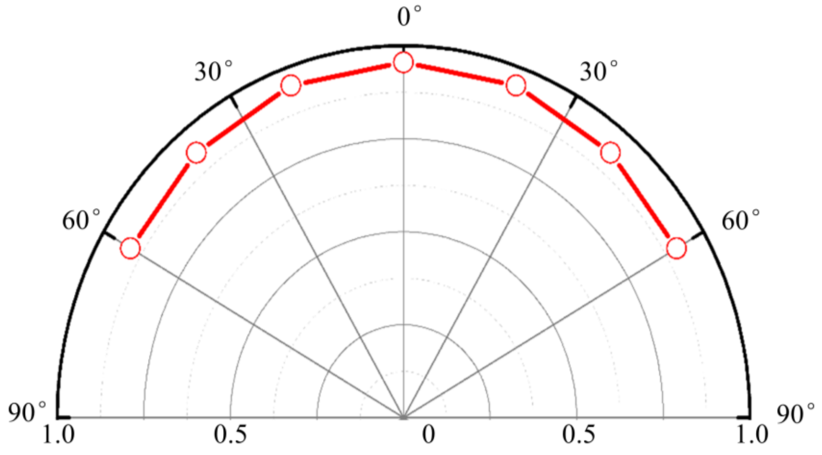

Figure S6 Directional emittance of the BRCC at different angles.

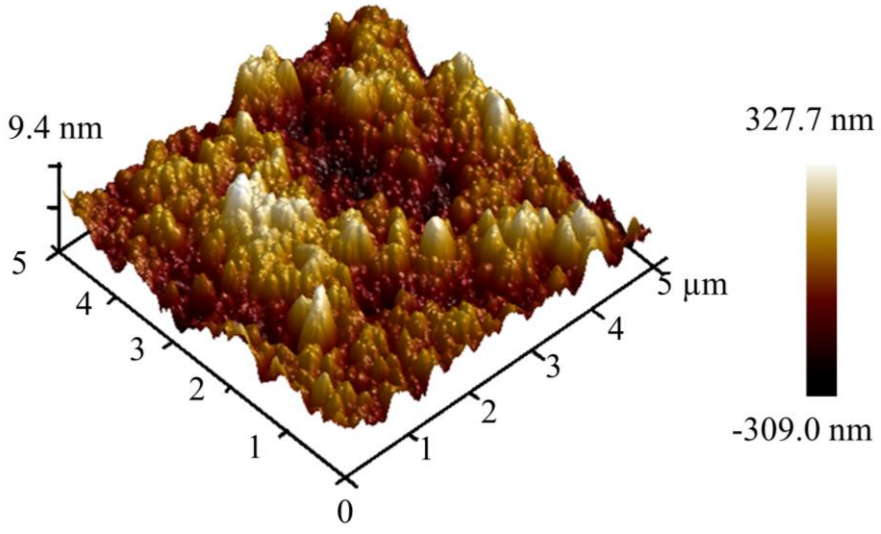

Figure S7 AFM images of the BRCC.

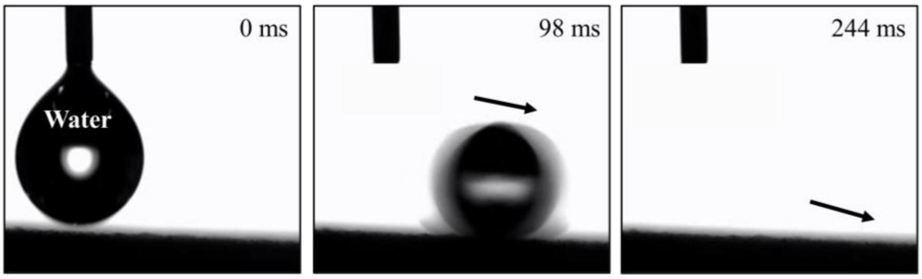

Figure S8 Water droplet rapidly rolls off the BRCC with a low sliding angle of ca. 3º.

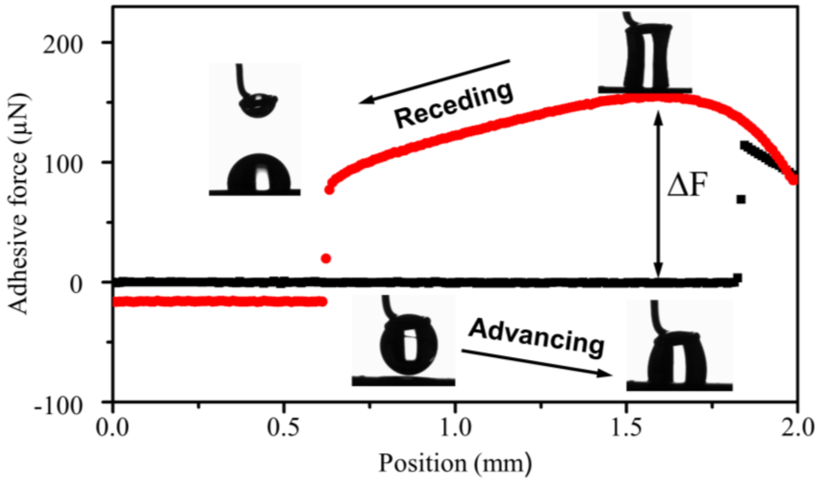

Figure S9 Force-distance curves and corresponding optical images of the FSi-coated Al alloy plate.

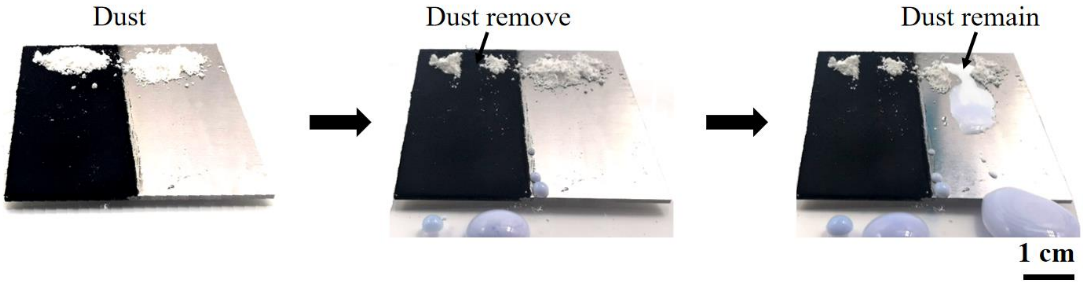

Figure S10 Comparison of surface dust removement *via* water flushing. After water flushing, dust was rapidly removed from the BRCC-coated Al alloy plate (black color). In contrast, dust still adhered on the bare one (silver color). Tilted angle was ca. 10°.

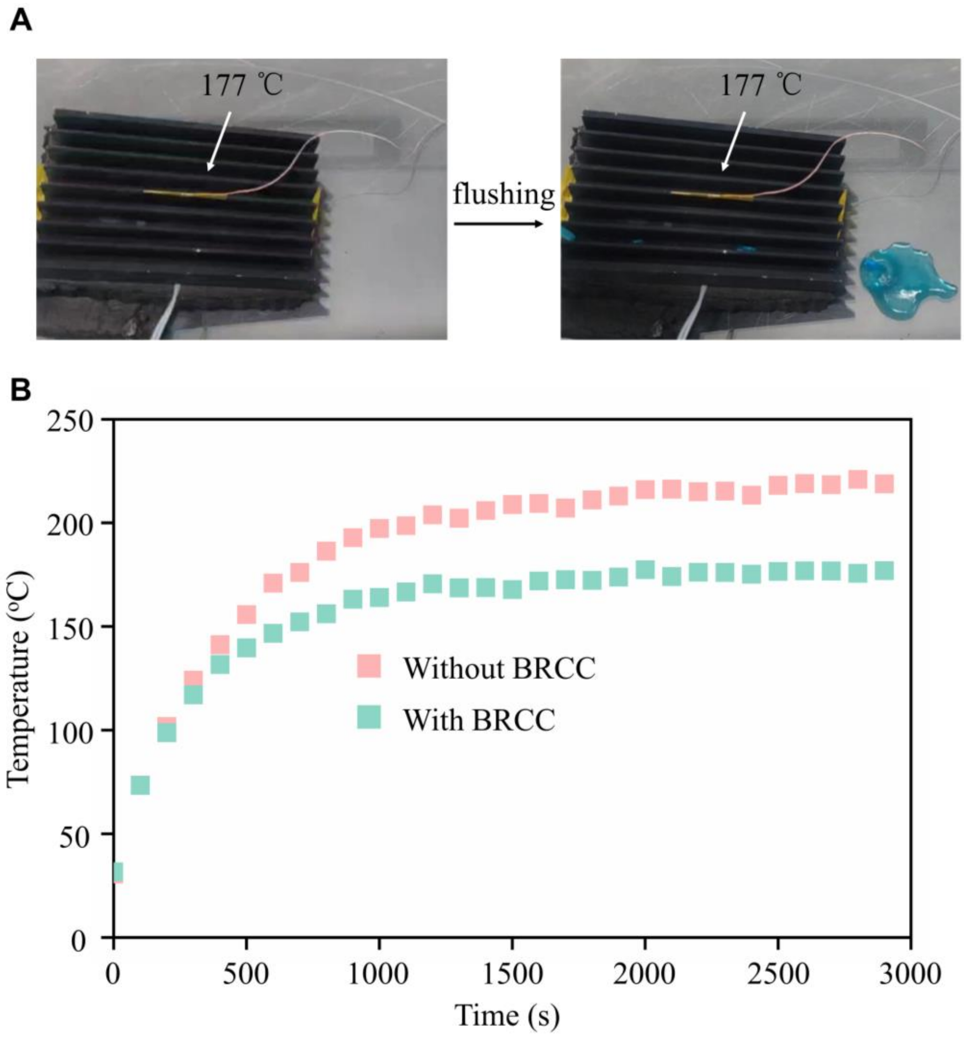

Figure S11 The high temperature resistance of the Al alloy heat sink with BRCC. A) The Al alloy heat sinks with BRCC could keep self-cleaning performance in high temperature. B) The Al alloy heat sinks with BRCC exhibited stable cooling performance in high temperature.

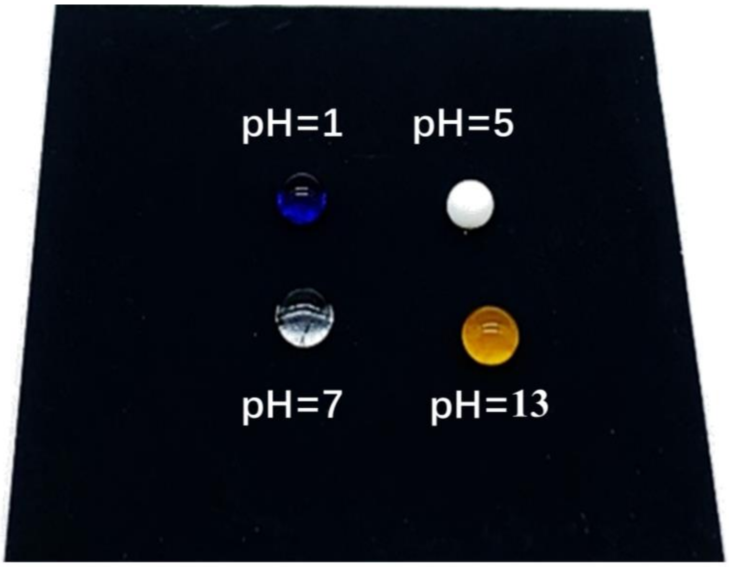

Figure S12 Chemical stability of the BRCC after dropping liquid droplets with different pH values.

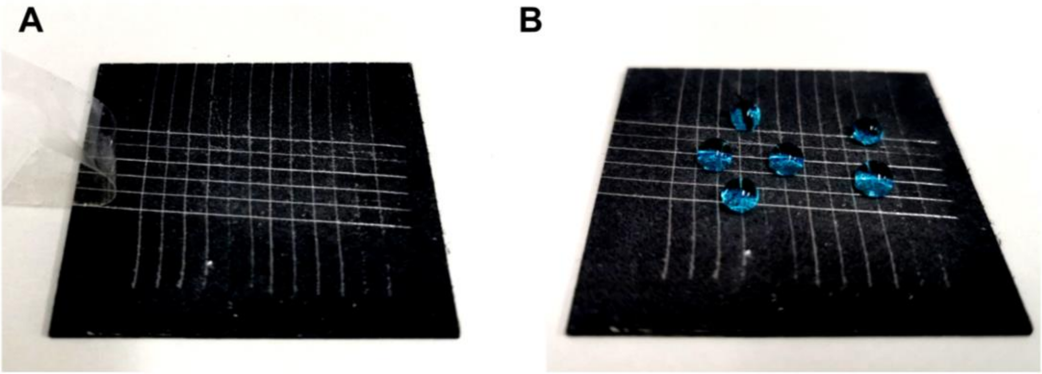

Figure S13 Robust wettability of the BRCC. A) Cross-cut test. B) Knife scratching test.

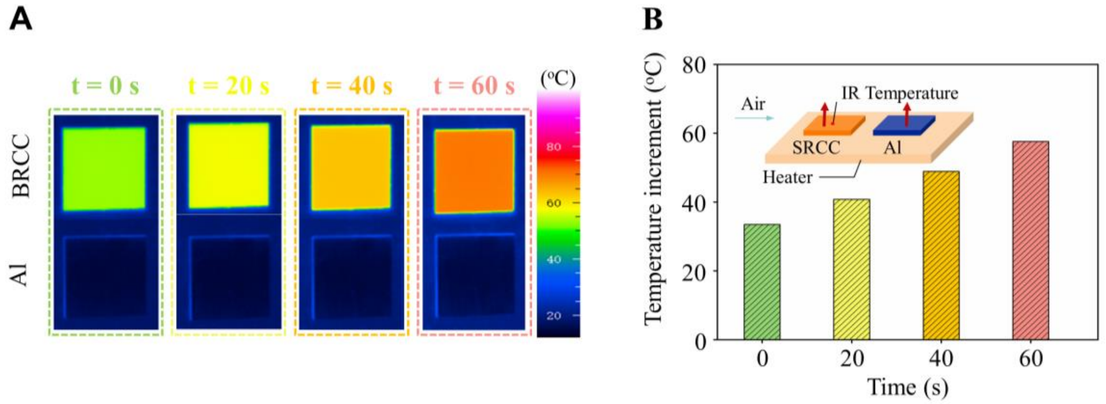

Figure S14 Comparison of thermal property between the BRCC-coated and bare Al alloy plates. A) Infrared images of the BRCC-coated and bare Al alloy plates after heating times from 0 to 60 s. B) Compared with Al alloy plate, the BRCC-coated one shows temperature increment with the increase of heating times.

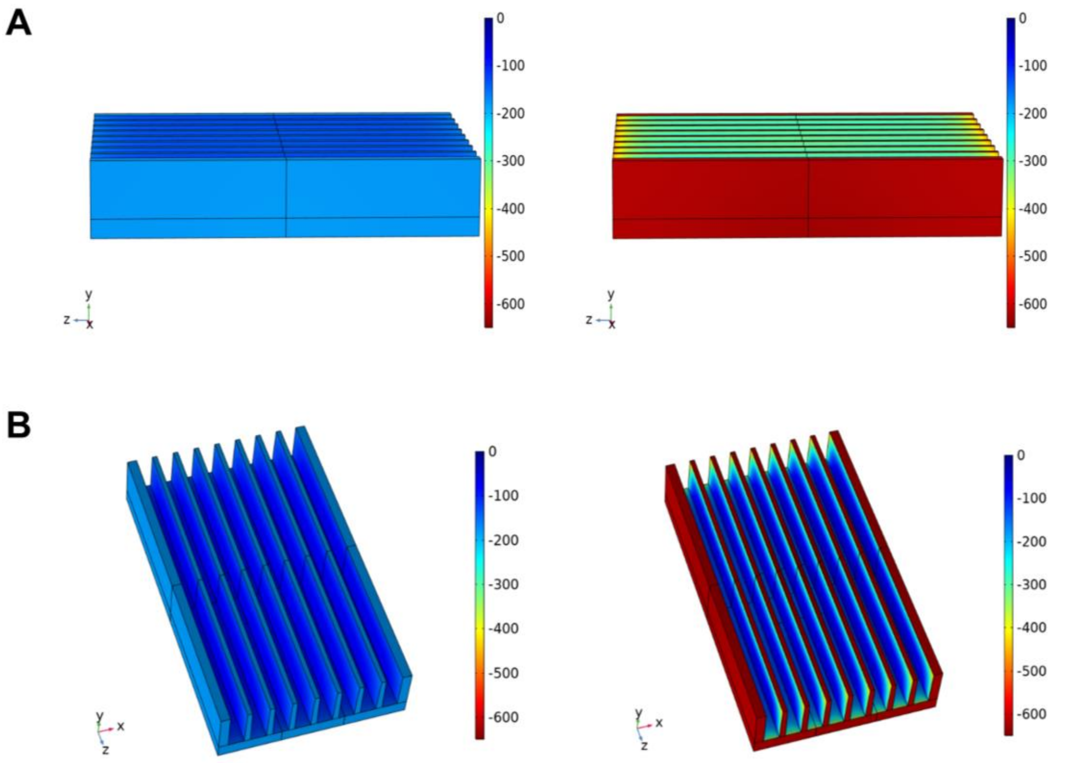

Figure S15 Simulation of radiative heat flux of heat sinks. A) The heat sink with (right) and without (left) the BRCC from subduction view at a heat power of 18 W. B) Side view.

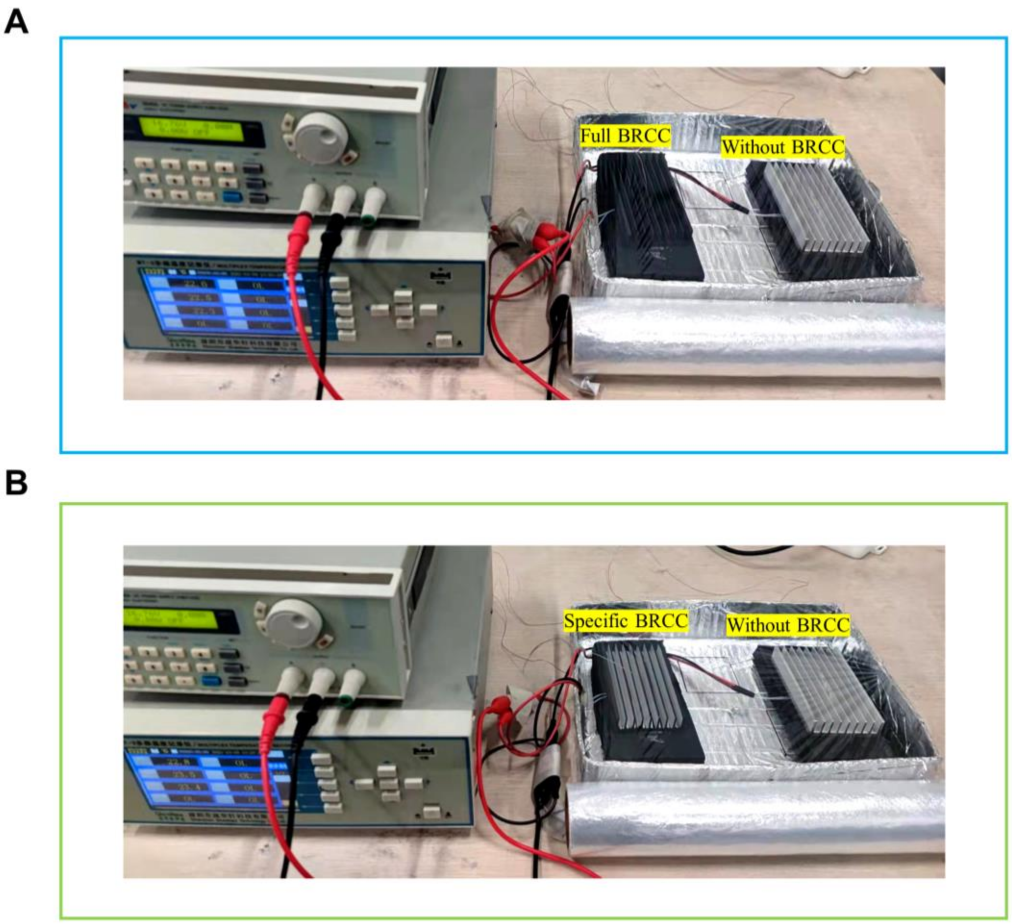

Figure S16 The experimental set-up for temperature measurement of Al alloy heat sink with full BRCC and specific BRCC. A) Al alloy radiator with full BRCC. B) Specific BRCC.

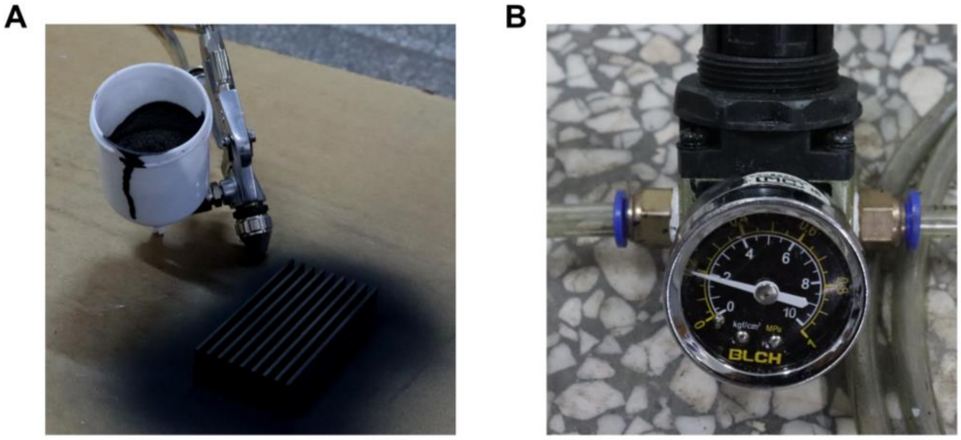

Figure S17 Spraying the BRCC on Al alloy heat sink. A) The image of spray gun and the Al alloy heat sink with the BRCC. B) The pressure for spraying BRCC (an air pressure of 0.2 MPa).

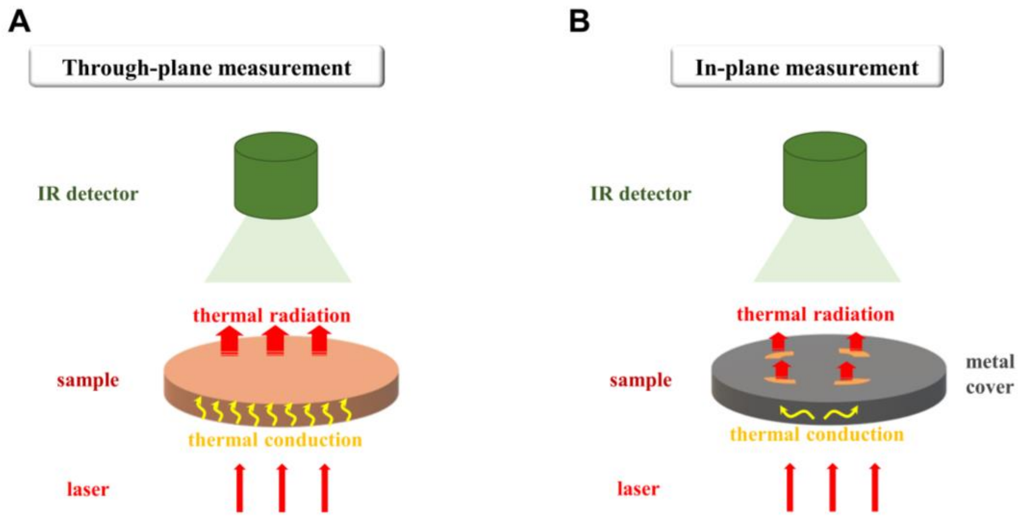

Figure S18 Schematic of the thermal diffusivity measurement process. A) Through-plane measurement. B) In-plane measurement.

3. Supplementary Table

Table S1 Simulation of heat transfer property for the heat sink under different heating powers.

Heating power (W)	Sample	Radiative Ratio (%)	Total heat transfer coefficient		Thermal resistance	
			Value (W/(m^2 K))	Increased Radio (%)	Value (W/k)	Decreased ratio (%)
10	Bare Al alloy	19.40	5.17	25.1	6.27	-19.5
	BRCC	40.20	6.47		5.05	
14	Bare Al alloy	19.17	5.61	24.7	5.79	-19.1
	BRCC	39.71	7.00		4.69	
18	Bare Al alloy	19.78	5.95	25.2	5.47	-19.3
	BRCC	39.89	7.46		4.42	

4. Supplementary Movies

Movie S1: Water droplet rolls off the BRCC-coated Al alloy plate.

Movie S2: Dynamic impact process of water droplet on the BRCC.

Movie S3: Dynamic impact process of water droplet on the bare Al alloy plate.

Movie S4: Viscous mud rolls off the BRCC-coated Al alloy heat sink.

Movie S5: Viscous mud adheres on the bare Al alloy heat sink.

Movie S6: Self-cleaning performance in high humidity.

Movie S7: Self-cleaning performance in high temperature.

Movie S8: Flushing process on the BRCC after 50-cycle abrasion.

Movie S9: Spraying the BRCC on Al alloy heat sink.
